# Supplementary material for: The challenge of involving old patients with polypharmacy in their medication during hospitalization in a medical emergency department: An ethnographic study
Source: PLoS One. 2021 Dec 30;16(12):e0261525. doi: 10.1371/journal.pone.0261525 (PMC8717970; doi:10.1371/journal.pone.0261525)
Supplement: S1 File — (DOC) [file pone.0261525.s001.doc]

**Interview guide: Medication in the Emergency Department / Emergency Clinic**

**Introduction**

Presentation and thank you for participating. Consent form is signed. The purpose of the research project is described:

**The aim of the Ph.D. project is** to qualify medication conversations in the Emergency Department / Emergency Clinic, by introducing joint decision-making with older poly-medicated patients. A communication tool that targets the involvement of older patients with polypharmacy is used, so that the patient's medication is chosen in accordance with the patient's preferences and values.

**The purpose of the interview is** to broaden the staff's perspective on patient involvement in the medication in an Emergency Department by elaborating on key themes that have been identified through field observations in the department. The focus is on barriers and facilitators.

**The framework (Formalia) is** "Confidential space" ensured anonymity, no right or wrong answers, not answering is fine, recording the interviews to remember what it said.

Time frame approx. 30 -45 min., interview guide.

The interview focuses on the main theme: Patient involvement in the medication in an Emergency Department and the people, materials, and systems that influence the process.

**A brief description of you and your employment.** State what I know and then ask about job title, terms of employment, length of employment, level of experience of dosing / prescribing / dispensing medication to patients.

**Then ask the questions below**

| Topic | Example of question | Follow-up question | Purpose of the question |
| --- | --- | --- | --- |
| 1. Medication in the e-health platform/SMC | How do you experience the medication process before and after introduction of the e-health platform/SMC?    (The medication process is everything from prescription, dosage, administration, patient information, documentation, etc.) | Do you think it has become easier or more difficult to involve the patients in the medication after introduction of the e-health platform?  (If you do not know about the e-health platform, describe how you have experienced the medication process in previous IT systems – elaborate) | Uncover the IT system's potential and barriers regarding patient involvement in the medication process, including the IT system's impact on staff's opportunities for patient involvement. |
| 1. Patient involvement in the medication process in the Emergency Department/Emergency Clinic | Do you involve patients in their medication?  Why/why not?  At what point in the process?  Why? | Are there any situations when the physician/pharmacist/nurse decide without involving the patient? | From the staff's perspective, identify which stages in the medication process in an Emergency Department are most relevant for involving the patients? |
| 1. Opportunities and barriers for patient involvement | Which are the biggest barriers in patient involvement in the medication process?  Which are the biggest opportunies? |  | In the field observations, several barriers for patient involvement of old poly-medicated patients were identified:  1) unmanageable medication that complicates patient involvement  2) patients' cognitive situation  3) old authoritative patients  4) when home care has taken over the medication, patients are difficult to engage  5) lack of time for communication in the Emergency Department  6) language differences or barriers between physicians and patients.  “Management of expectations" is advisable: "The patients does not speak up unless we ask them".  Involving old patients in the medication is complex.  **The potentials were:**  1) patients should be aware of what medication they receive  2) most old patients WANT to be involved in the medication process but can find it difficult for various reasons  3) involving the relatives whenever possible: “The relatives are in control” |
| 1. Unmanageable medication | What is meant by the term “unmanageable” medication?  Can you elaborate? | Is it about the process, the interdisciplinary collaboration, the patient, professional understanding, or something else? | In the field observation, the staff complains about the patients' "unmanageable medication" and "difficulties to get an overview". What does that imply? |
| 1. Is there a connection between "unmanageable medication" and patient involvement? | Do you see any connections between "unmanageable medication" (or the number of drugs) and the possibility of patient involvement? |  | In the field observations, the doctors, pharmacists, and pharmaconomists spent a long time getting an overview of the patients' medication in the IT-systems and they often complained that it was difficult to get an overview. Do they try harder or less to involve the patients due to this? |
| 1. The medication list | Do you use the medication list when you see the patients?  Why/why not?  In which situations will you use it/not use it? | Which advantages/disadvantages do you see in using the medication list? | Elaborate on different perspectives on the use of the medication list during medication interviews (In the field observations, I saw that the staff usually do not involve the medication list to discuss medication with the patient.) |
| 1. Recognized and unrecognized roles in the medication process | How much time do the patients' medication take up in the Emergency Department/Emergency Clinic?  Elaborate. | Do you find this important?  Optional question: Why do you think that some healthcare professionals don’t think they are part in the medication process? | I ask because in the field observations, I heard staff members say that they were not involved in the medication process though the field observations showed that they were.  What could be the reason? |
| 1. The discharges | What does it entail when the ED- physicians distinguish between medication concerning acute problems and medication prescribed by the General practitioner, in which they would not interfere?  Does it cause problems?  If so, in which way? | Optional question: What do you think about the terms “acute problem” or “General practitioner problem”? | The field observations showed that the physicians often distinguished between acute medication problems and General practitioner problems and most physicians preferred not to interfere with General practitioner medication problems. This is a dilemma for ED physicians.  How do we solve the problem?  Who is responsible for the patients' medication?  How does this affect the patient involvement? |
| 1. Completion | Do you have anything else on your mind?  Is there anything else you think we need to know? |  |  |

Completion:

Five minutes informal conversation. Ask if the informant has any questions. End the interview by thanking for the participation.
