# Supplementary material for: The challenge of involving old patients with polypharmacy in their medication during hospitalization in a medical emergency department: An ethnographic study
Source: PLoS One. 2021 Dec 30;16(12):e0261525. doi: 10.1371/journal.pone.0261525 (PMC8717970; doi:10.1371/journal.pone.0261525)
Supplement: Attachment — Submitted filename: Response to reviewers.doc [file pone.0261525.s003.doc]

Hvidovre, th. 29. nov. 2021

**Response to Editor and Reviewers**

Manuscript: PONE-D-21-12087R1

”*The challenge of involving old patients with polypharmacy in their medication during hospitalization in a medical emergency department: An ethnographic study*”

Dear

Adam Todd, PhD
Academic Editor
PLOS ONE

Thank you very much for your and the reviewers' excellent response to our paper and for providing us with the opportunity to improve it. Please read the text box below for our responses to reviewer 1's comment.

Indeed, we do belive that socioeconomic disparity also is a rising concern in Denmark (as it is in other countries). We are fully aware of the challenges that multimorbidity patients from low-income families face when attempting to engage in treatment decisions.

However, we do also believe that patients with inadequate health literacy still may have treatment preferences, eventhough they have difficulties in communicating with their physicians or pharmacist. Focusing on the patients preferences is an essential part of SDM and in this light, we hope to engage more multimoribidity patients in the medical treatment and health with SDM approach.

As requested, we also went over the reference list to confirm that none of the references are retracted. However, we have removed two references in our revised manuscript: 1) “*Høj K, Mygind A, Livbjerg S, Bro F. Deprescribing of inappropriate medication in primary care. Ugeskrift for læger. 2019.* And 2) *Charles C, Gafni A, Whelan T. Decision-making in the physician-patient encounter: revisiting the shared treatment decision-making model. Soc Sci Med. 1999.* We remowed the two references primarily because their content was similar to that of others references in the reference list, and to make room for two new references recommended by reviewer 1, which we have included in the disussion section to support the argument that people with lower socioeconomic baggrounds find it more difficult to participate in their treatment.

We hope that you will find our revised manuscript suitable for publication in PLOS ONE.

Yours Sincerely,

On behalf of the authors

Pia Keinicke Fabricius, PhD student

Clinical Research Center (Section 056)

Amager Hvidovre Hospital, University of Copenhagen

Kettegaard Allé 30

2650 Hvidovre, Denmark

Email: [pia.keinicke.fabricius@regionh.dk](mailto:pia.keinicke.fabricius@regionh.dk)

Phone +45 51845939

| **Editor and reviewer feed-back: Our answers** | |
| --- | --- |
| **Editor:** Reviewer 1 makes an important role in acknowledging the role of the social determinants in shared decision-making, and how this is potentially more challenging for people of lower socioeconomic status.  I invite you to reflect on this comment, and perhaps acknowledge this in the discussion of your work. | See our reflection in the introduction section of this letter.  We have also added text in the discussion section. Se our answer to reviewer 1 in section 6. |
| 1. If the authors have adequately addressed your comments raised in a previous round of review and you feel that this manuscript is now acceptable for publication, you may indicate that here to bypass the “Comments to the Author” section, enter your conflict of interest statement in the “Confidential to Editor” section, and submit your "Accept" recommendation.  Reviewer #1: All comments have been addressed  Reviewer #2: All comments have been addressed | We are glad that you think so |
| 2. Is the manuscript technically sound, and do the data support the conclusions?  Reviewer #1: Yes  Reviewer #2: Yes | We are glad that you think so |
| 3. Has the statistical analysis been performed appropriately and rigorously?  Reviewer #1: N/A  Reviewer #2: N/A |  |
| 4. Have the authors made all data underlying the findings in their manuscript fully available?  Reviewer #1: Yes  Reviewer #2: Yes |  |
| 5. Is the manuscript presented in an intelligible fashion and written in standard English? Reviewer #1: Yes  Reviewer #2: Yes | We are glad that you think so |
| 6. Review Comments to the Author  Reviewer #1: This is a much improved second submission. The authors have worked very hard to address all the reviewers comments from the first submission, and the article is now almost ready to be accepted. I would recommend one more round of minor revisions to address a topic that is hugely important in the literature on multi-morbidity, which is social inequality or, more broadly, social determinants. The most cited study on multi-morbidity, Barrett et al. 2012 (doi: 10.1016/S0140-6736(12)60240-2) focuses heavily on the role of social determinants in relation to onset and severity of multi-morbidity, making the crucial observation that social deprivation can shift the onset of multi-morbidity foreword by 10 or 15 years. Social deprivation also has ramifications for shared decision-making, because patients from poor backgrounds find participation in decision-making so much harder, on so many levels. This has also been shown by several qualitative studies, e.g. recently by Ecks 2021 (<https://link.springer.com/article/10.1007/s11013-020-09699-x>). Since the focus of this article is on shared decision-making, at least a few lines should be devoted to the role of social determinants. It is quite possible that Denmark has less social inequality than other countries, and that therefore social determinants do not play a big role in this context, but even there I would have expected some discussion of it.  Reviewer #2: Thank you to the authors for addressing my comments so thoroughly. I recommend this paper for publication. | Reviewer 1:  Thank you very much for raising this important aspect and challenge in relation to SDM.  We agree with you, and have added the following text in **the discussion section at page 28 bottom- section and page 29 top- section.**    Therefore, it is essential for our society (and patients) that vulnerable patients with multimorbidity and polypharmacy be empowered to better care for themselves.  *“However, at the same time, Ecks' [2021] recent study has shown that socioeconomic hardship and inequality make it more difficult for people from disadvantaged backgrounds to engage actively in treatment decision-making [43]. The importance of social inequality in multimorbidity is emphasized further in Barrett et al.‘s study on multimorbidity, which reveals that the onset of multimorbidity occurs ten to fifteen years earlier in socially deprived areas, calling into question the existing single disease treatment approach in healthcare [44]*”*.*    Thank you, reviewer 2. |
| 7. PLOS authors have the option to publish the peer review history of their article ([what does this mean?](https://journals.plos.org/plosone/s/editorial-and-peer-review-process#loc-peer-review-history)). If published, this will include your full peer review and any attached files  **Do you want your identity to be public for this peer review?** For information about this choice, including consent withdrawal, please see our [Privacy Policy](https://www.plos.org/privacy-policy).  Reviewer #1: No  Reviewer #2: **Yes:** Nina Fudge  . | Thank you very much |
